# Safety and effectiveness of remote monitoring and prioritization of patients awaiting transcatheter aortic valve implantation: a propensity-matched prospective observational cohort study

**DOI:** 10.1093/ehjdh/ztaf114

**Published:** 2025-10-03

**Authors:** Mihir A Kelshiker, Karanjot Chhatwal, Patrik Bachtiger, Emily Jin, Josephine Mansell, Olivia Molloy, Mee-Keah Tang, Sarah Pearse, James Bird, Joshua March, Chris Robbins, Saloni Nakhare, Amanda Varnava, Ramzi Khamis, Adam Hartley, Saud Khawaja, Neil Ruparelia, Nearchos Hadjiloizou, Sayan Sen, Ghada Mikhail, Iqbal S Malik, Nicholas S Peters

**Affiliations:** National Heart and Lung Institute, Imperial College London, Hammersmith Hospital, Du Cane Road, London W12 0HS, UK; Imperial College Healthcare NHS Trust, The Bays, South Wharf Road, London W2 1NY, UK; National Heart and Lung Institute, Imperial College London, Hammersmith Hospital, Du Cane Road, London W12 0HS, UK; Imperial College Healthcare NHS Trust, The Bays, South Wharf Road, London W2 1NY, UK; National Heart and Lung Institute, Imperial College London, Hammersmith Hospital, Du Cane Road, London W12 0HS, UK; Imperial College Healthcare NHS Trust, The Bays, South Wharf Road, London W2 1NY, UK; National Heart and Lung Institute, Imperial College London, Hammersmith Hospital, Du Cane Road, London W12 0HS, UK; National Heart and Lung Institute, Imperial College London, Hammersmith Hospital, Du Cane Road, London W12 0HS, UK; Imperial College Healthcare NHS Trust, The Bays, South Wharf Road, London W2 1NY, UK; Imperial College Healthcare NHS Trust, The Bays, South Wharf Road, London W2 1NY, UK; Imperial College Healthcare NHS Trust, The Bays, South Wharf Road, London W2 1NY, UK; Imperial College Healthcare NHS Trust, The Bays, South Wharf Road, London W2 1NY, UK; Imperial College Healthcare NHS Trust, The Bays, South Wharf Road, London W2 1NY, UK; Imperial College Healthcare NHS Trust, The Bays, South Wharf Road, London W2 1NY, UK; Imperial College Healthcare NHS Trust, The Bays, South Wharf Road, London W2 1NY, UK; National Heart and Lung Institute, Imperial College London, Hammersmith Hospital, Du Cane Road, London W12 0HS, UK; National Heart and Lung Institute, Imperial College London, Hammersmith Hospital, Du Cane Road, London W12 0HS, UK; Imperial College Healthcare NHS Trust, The Bays, South Wharf Road, London W2 1NY, UK; National Heart and Lung Institute, Imperial College London, Hammersmith Hospital, Du Cane Road, London W12 0HS, UK; Imperial College Healthcare NHS Trust, The Bays, South Wharf Road, London W2 1NY, UK; National Heart and Lung Institute, Imperial College London, Hammersmith Hospital, Du Cane Road, London W12 0HS, UK; Imperial College Healthcare NHS Trust, The Bays, South Wharf Road, London W2 1NY, UK; National Heart and Lung Institute, Imperial College London, Hammersmith Hospital, Du Cane Road, London W12 0HS, UK; Imperial College Healthcare NHS Trust, The Bays, South Wharf Road, London W2 1NY, UK; National Heart and Lung Institute, Imperial College London, Hammersmith Hospital, Du Cane Road, London W12 0HS, UK; Imperial College Healthcare NHS Trust, The Bays, South Wharf Road, London W2 1NY, UK; Imperial College Healthcare NHS Trust, The Bays, South Wharf Road, London W2 1NY, UK; National Heart and Lung Institute, Imperial College London, Hammersmith Hospital, Du Cane Road, London W12 0HS, UK; Imperial College Healthcare NHS Trust, The Bays, South Wharf Road, London W2 1NY, UK; National Heart and Lung Institute, Imperial College London, Hammersmith Hospital, Du Cane Road, London W12 0HS, UK; Imperial College Healthcare NHS Trust, The Bays, South Wharf Road, London W2 1NY, UK; National Heart and Lung Institute, Imperial College London, Hammersmith Hospital, Du Cane Road, London W12 0HS, UK; Imperial College Healthcare NHS Trust, The Bays, South Wharf Road, London W2 1NY, UK; National Heart and Lung Institute, Imperial College London, Hammersmith Hospital, Du Cane Road, London W12 0HS, UK; Imperial College Healthcare NHS Trust, The Bays, South Wharf Road, London W2 1NY, UK

**Keywords:** Digital health, Aortic stenosis, Remote patient monitoring, Transcatheter aortic valve implantation

## Abstract

**Aims:**

Health systems face increasing waiting times for transcatheter aortic valve implantation (TAVI), incurring excess deaths and morbidity. To determine whether remote patient monitoring (RPM) using connected technologies can mitigate these risks by prioritizing patients awaiting TAVI, we aimed to measure the clinical safety and effectiveness of an RPM-based prioritization programme.

**Methods and results:**

Prospective observational cohort study of all patients awaiting TAVI at Imperial College Healthcare NHS Trust, London, UK, between 24th April 2023 and 15th November 2023. An RPM pathway was implemented for all patients accepted for TAVI. These patients responded to a weekly symptom questionnaire via web, smartphone RPM platform or telephone monitoring; with rule-based clinical escalation. The primary endpoint was the rate of adverse events (defined as emergency department presentation, unplanned hospitalization, or death), compared with a propensity score-matched (PSM) historical control group. Secondary endpoints included pathway performance characteristics for detection of deterioration. 200 patients met inclusion criteria. Despite growth of the waiting list, responsible for longer waiting times experienced by the RPM group [median 104 days (IQR 61.00–176.00) vs. 75 days (IQR 38.75–118.00)], there was no difference in rates of adverse events between RPM-patients and historical controls (Log rank *P* = 0.9). The RPM pathway had high sensitivity for prediction of waiting list death (100%). Patients deemed at high-risk of deterioration experienced shorter waiting times to treatment.

**Conclusion:**

RPM for patients awaiting TAVI is feasible and may mitigate the adverse effects of longer waiting times through accurate detection of deterioration and by informing prioritization decisions.

## Graphical Abstract

**Graphical Abstract:**
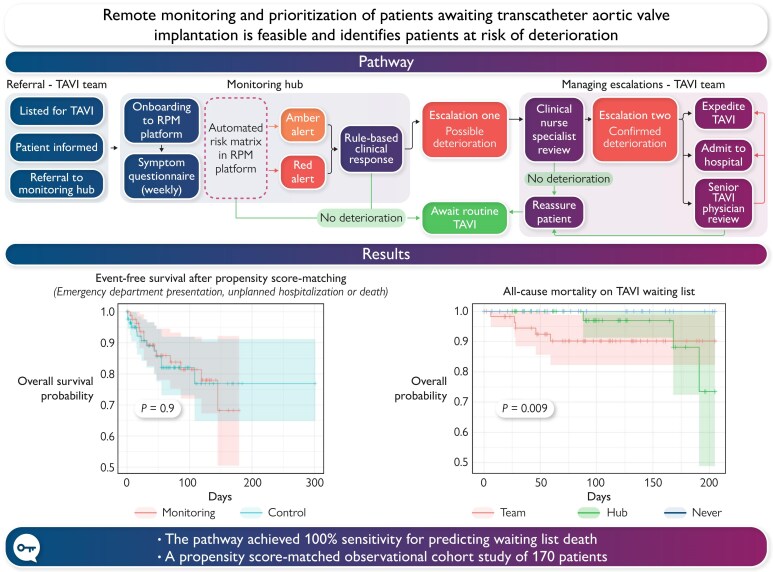

## Introduction

Waiting times for transcatheter aortic valve implantation (TAVI) are increasing globally.^1,2^ Although there is a paucity of data to support an acceptable waiting time, it is estimated from Canadian registry data that there is a cumulative incidence of death up to 4.3% at 100 days.^1^ Furthermore, longer waiting times are associated with higher waiting list morbidity (e.g. from heart failure hospitalization), and cost from unplanned episodes of care.^1,3^ Performing emergency TAVI in patients who have deteriorated clinically to the point of heart failure hospitalization incurs greater intra- and post-procedural complications and complexity,^4^ with knock-on cancellation and net reduction in capacity for elective procedures, ultimately undermining the cost-effectiveness of a TAVI programme.^3^ Procedural waiting times at UK regional TAVI centres are not routinely reported, however they are invariably longer than the recommended national upper limit of 4 weeks,^5^ with a growing proportion of TAVIs performed as urgent or emergency procedures.^2^ Mitigating risk from longer waiting times is therefore a priority in low-capacity settings relative to the needs of the population.

Remote patient monitoring (RPM) using digital health technologies can improve clinical and health economic outcomes in patients with implantable cardiac devices,^6^ heart failure^7,8^ and acute coronary syndromes.^9^ Longitudinal, data-driven monitoring strategies may support precise and timely identification and prioritization of patients at risk of deterioration on the TAVI waiting list.

In this study, we aimed to test the hypotheses that implementing a DHT-enabled remote monitoring and prioritization pathway for patients on the TAVI waiting list is feasible, safely reduces the risk of waiting list death in the context of escalating waiting times, can effectively identify patients at risk of waiting list deterioration, and therefore inform prioritization decisions.

## Methods

### Study design

We used a prospective, observational cohort study design following the Strengthening the Reporting of Observational Studies in Epidemiology (STROBE) checklist^10^ (see Supplementary material online, *Table S1*).

### Inclusion criteria

This study involved all patients awaiting TAVI at Imperial College Healthcare NHS Trust (ICHNT) between 24th April and 15th November 2023. All patients had been assessed by the TAVI team, had accepted the offer of a TAVI, and were awaiting a TAVI procedural date. These dates were selected to align with the start of the RPM programme and to allow a minimum of 6 months of follow-up for the initial cohort of patients.

### Exclusion criteria

We excluded patients who, during the study period, were removed from the waiting list; had received their TAVI prior to the start of remote monitoring; changed treatment centre; were listed for a different aortic procedure or refused digital or telephone-based remote monitoring. Reasons for removal from the waiting list included patients becoming uncontactable despite repeated attempts by the clinical team; a revised patient decision to decline TAVI; or a reassessment of a patient’s clinical risk-benefit profile as unfavourable due to changes in health status (e.g. advancing frailty).

### Intervention

**Figure 1:**
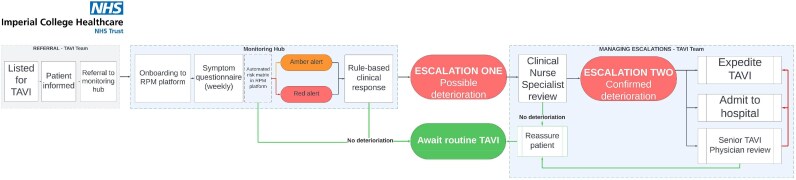
Flow diagram of the pathway for remote monitoring and prioritization of patients on the waiting list for TAVI. Left to right; patients are referred for monitoring by the TAVI team—consisting of the TAVI Clinical Nurse Specialists and the Senior TAVI Physician(s). The monitoring hub team onboard the patient to the RPM platform and encourage adherence to weekly symptom questionnaires. Week-on-week deterioration is escalated by the monitoring hub team to the TAVI CNS, who can take appropriate clinical action. This includes escalation for patient review by a Senior TAVI physician. RPM, remote patient monitoring.

At a single time-point (24th April 2023), all patients on the TAVI waiting list at ICHNT were instructed to register with an online RPM platform (Ortus iHealth, Ortus Solutions Limited, UK) as part of their usual care. All patients prospectively added to the waiting list between 24th April 2023 and 15th November 2023 was also instructed to do so. Participants received a postal information leaflet informing them of the RPM pathway. The RPM platform was accessible as a web app through a computer or laptop internet browser, or via a downloadable smartphone application (*Figure 1*). Patients received a postal information sheet to guide registration and were also supported to complete this via follow-up telephone calls from the NHS North West London Virtual Hospital (NWLVH) team. NWLVH is a team of general nurses, who are physically co-located in a monitoring ‘hub’. They operate DHT-enabled clinical pathways on behalf of specialist teams, in accordance with mutually agreed standard operating procedures that include clear routes for rule-based clinical escalation and responsiveness. The NWLVH team is also responsible for maximizing patient digital inclusion and engagement through provision of telephone and video-based technical support. Patients who could not complete registration despite graded support from NWLVH received weekly telephone-based monitoring. We have previously published the graded support system and criteria for receiving telephone-based monitoring.^11^ In short, telephone-based monitoring was offered to patients who did not have access to a smartphone with mobile data, no access to a computer with internet access, and were not visited at least once per week by someone who could support them to access the platform.

Participants were instructed to complete a symptom questionnaire (see Supplementary material online, *Figure S1*) via the platform on a weekly basis, and more frequently if their symptoms deteriorated. Patients who could not manage remote monitoring technology with support were offered telephone-based symptom monitoring by the NWLVH team using the same questionnaire. All participants were reminded to complete the weekly questionnaire via SMS. Participants were informed that questionnaire responses were not reviewed in real-time, and were instructed to seek urgent medical attention via 111 or 999 in the event of an emergency.

The patient symptom questionnaire and corresponding response risk matrix (see Supplementary material online, *Figure S1*) were developed through expert consensus meetings between eight lead clinicians in the TAVI team at ICHNT. Responses triggering amber or red on the risk matrix were actioned by the NWLVH team, with protocolized clinical escalation routes to the TAVI Clinical Nurse Specialists (CNS), who are the primary clinical and operational contact point for the TAVI service (clinical valve coordinator).

Red responses required clinical review by the NWLVH team within 24 h. Amber responses required clinical review within 48 h. The NWLVH examined symptom reports, following a rule-based approach to identify any week-on-week deterioration in the symptom responses. Patients meeting these criteria were escalated to the TAVI CNS via telephone, email and weekly virtual team meetings (escalation one).

The TAVI CNS could then conduct their own remote clinical review (e.g. by video or telephone call), and, based on their clinical judgement, could either reassure the patient, or further escalate care (escalation two). Escalation two involved one of three actions (in increasing order of urgency):

1. Schedule an urgent outpatient clinic review with a Senior TAVI Physician
2. Schedule an expedited TAVI procedural date
3. Admit the patient to hospital

The receipt of each inbound symptom questionnaire was acknowledged with a message from the NWLVH team to the patient, delivered via the RPM platform.

### Control group

A control cohort was identified from the TAVI waiting list immediately predating the implementation of the remote monitoring programme. Unselected patients who had received TAVI were chronologically identified prior to 24th April 2023. This index date was chosen to eliminate overlap with the intervention cohort, and method of patient selection chosen to minimize differences in departmental policy and procedural capacity between RPM and control groups (given these are important contextual factors that are difficult examine quantitatively as covariates).

### Data collection

For both RPM and control patients, we extracted baseline demographics and clinical characteristics from the primary electronic health record (Cerner, USA). Where available, we recorded Systematized Nomenclature of Medicine Clinical Terms (SNOMED-CT) and ICD-10 codes for diagnoses and comorbidities. We examined the London Care Record (LCR), a pooled, live clinical dataset of all NHS care providers in London to record frequency and location of emergency department presentations, unplanned hospital admissions and mortality on the waiting list.

### Outcomes

The primary outcome was major adverse events, defined as ED presentation not resulting in admission, unplanned inpatient hospitalization (including admissions via ED or direct transfers) or all-cause death whilst on the waiting list for TAVI. Secondary outcomes are summarized in *Table 1*.

**Table 1:** Secondary outcomes and definitions

Secondary outcome	Definition
Waiting time	Days between being placed on the waiting list for TAVI and procedure completion
(RPM cohort only) Digital inclusion	Completion of at least one symptom questionnaire via the online platform
(RPM cohort only) Performance metrics for detecting deterioration (sensitivity, specificity, positive and negative predictive value)	Two definitions of deterioration considered for each escalation point (*Figure 1*): Prediction of waiting list death Prediction of unplanned hospitalization

### Bias

We reported differences in baseline characteristics between groups to identify possible selection bias in implementation of RPM.

### Statistical analysis

Data was summarized as mean (SD) or median (interquartile range) for skewed data. Comparisons between groups included two-sample *t*-tests for continuous variables, and χ^2^ tests for binary and categorical variables.

For both groups we measured unadjusted risk (cumulative incidence) of the primary endpoint. We created a univariate logistic regression model to identify any association between waiting time and adverse events. A further logistic regression model was created with waiting times segmented into 50-day increments. These analyses were limited to the historical control cohort to minimize the risk of confounding from partial exposure to the intervention, with univariate analysis undertaken to avoid over-fitting.^12^ In the full cohort (*n* = 36 events), an exploratory multivariable logistic regression adjusting for intervention/control allocation and up to three covariates was performed to identify an association between waiting time and MACE.

We performed multivariable logistic regression modelling to quantify any demographic determinants of digital inclusion, including age, sex and ethnicity as covariates, with the dependent variable being the completion of at least one symptom questionnaire via the online platform.

To account for differences in the baseline characteristics between groups, we performed propensity score matching (PSM). This enabled presentation of results in unitary terms that can more directly inform organizational policy (e.g. waiting time in days), compared with the more probabilistic terms derived from traditional multivariate logistic regression modelling.

To construct the PSM cohort, we included all historical controls and only those patients who were prospectively added to the TAVI waiting list after the start of the RPM programme. We excluded patients who were already on the TAVI waiting list on the 24th April 2023 from the PSM cohort, to mitigate the possible confounding effects of partial exposure to RPM. To construct the PSM cohort, we applied 1:1 nearest neighbour matching with a calliper width of 0.1 to the following covariates: age, sex, ethnicity, presence of ischemic heart disease, hypertension, chronic kidney disease, diabetes mellitus and active or previous malignancy. Echocardiographic and electrocardiographic variables were not included in the PSM model due to >10% missingness of data (see Supplementary material online, *Table S2*). We performed Kaplan-Meier analysis to measure differences in event-free survival between groups. Patients who received TAVI during the observation period were right-censored at the time of the procedure. As a sensitivity analysis, inverse probability of treatment weighting (IPTW) was performed using all eligible patients and an identical covariate set to the PSM model, with Kaplan-Meier analysis also performed for this cohort.

To measure the potential confounding effect of excluding a wider covariate set on the primary endpoint, a further sensitivity analysis was performed whereby missing data were addressed using multiple imputation by chained equations. Variables with >30% missingness or limited incremental value for confounding control (e.g. peak aortic velocity) were excluded to avoid unstable imputation and extreme weights. For echocardiographic parameters, LVEF, peak aortic valve gradient and dimensionless index were retained as robust, consistently recorded markers of function and stenosis severity.

To determine the performance characteristics of each escalation point for prediction of waiting list death and unplanned hospitalization, we examined the highest grade of clinical response prior to the event of interest (i.e. in the case of one patient having an unplanned hospitalization preceded by several symptom questionnaires that triggered red or amber alerts, the most urgent clinical response was considered).

Analyses used a time-to-first-event framework. Where an ED presentation culminated in an inpatient admission within the same episode, the event was classified as unplanned hospitalization and not additionally counted as an ED presentation. Death was counted irrespective of preceding events. Consequently, each participant contributed at most one event to the Kaplan–Meier analysis. For logistic regression models, the outcome was binary (≥1 event vs. 0); therefore, no patient contributed multiple events to the model.

## Results

### Patient characteristics

**Figure 2:**
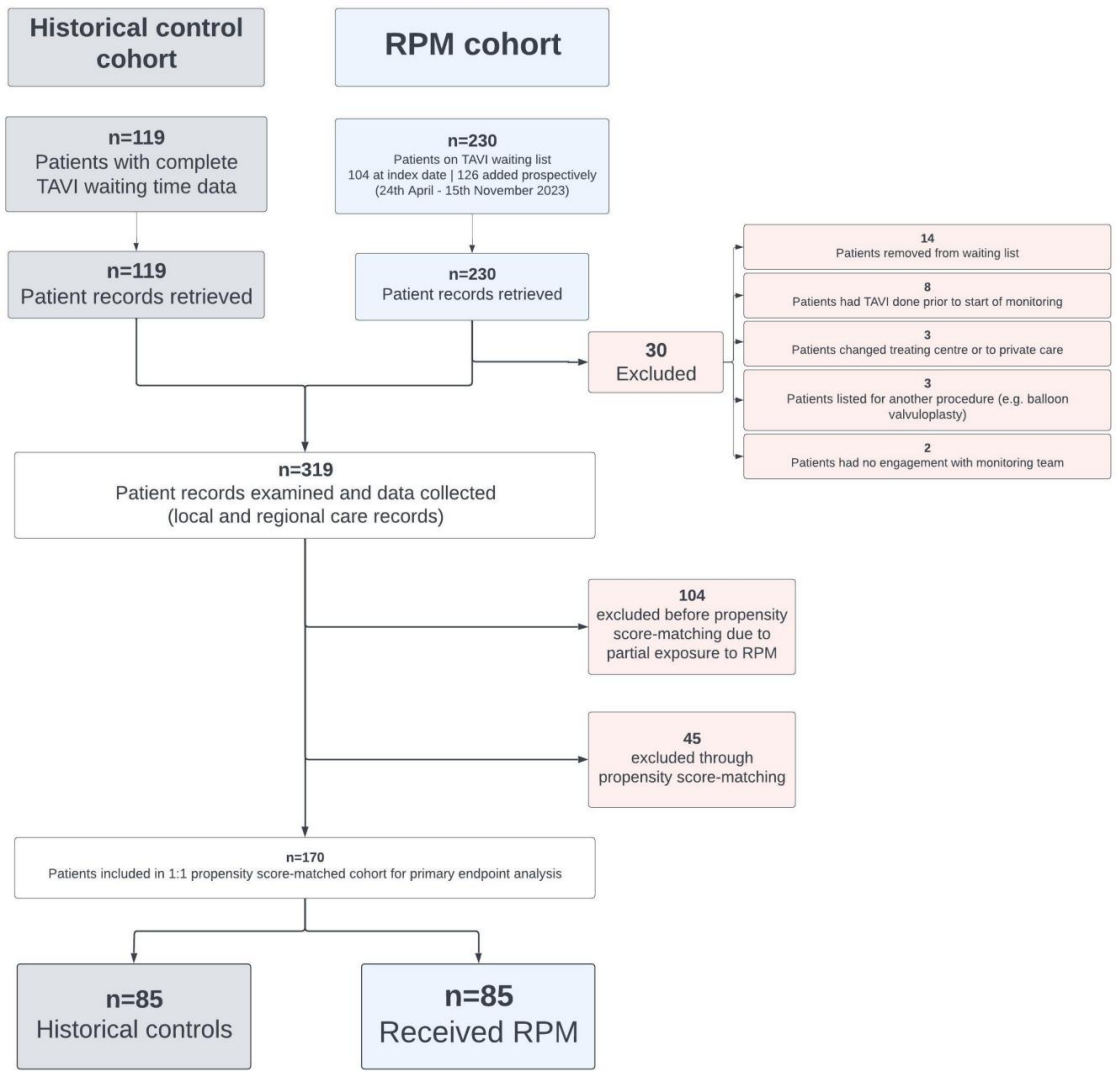
Patient flow through the study. Only patients added to the waiting list after 24th April 2023 were eligible for PSM to mitigate partial RPM exposure bias. ICHNT, Imperial College Healthcare NHS Trust.

We retrieved and examined records for a total of 230 patients who were on the TAVI waiting list between 23rd April and 15th November 2023. Patient flow through the study is described in *Figure 2*. A total of 200 patients met the inclusion criteria. The records of 119 consecutive historical control patients who had received TAVI at ICHNT prior to the observation period were examined. This was the maximum number of consecutive patients with complete TAVI referral and waiting time data availability, due to departmental IT changes.

Patient demographic, clinical characteristics and unadjusted outcomes stratified by group are presented in *Table 2*. At baseline, the RPM and historical control cohorts were well matched by age, sex, comorbidities, and echocardiographic parameters. A greater proportion of patients in the RPM group were of White ethnicity (37.5% vs. 24.4%, *P* = 0.02). There was a higher prevalence of cognitive impairment in the RPM cohort (0% vs. 5.5%, *P* = 0.008).

**Table 2:** Patient characteristics by group

	Control (*n* = 119)	RPM (*n* = 200)	*P*-value^a^
**Demographics**			
Age (SD)	80.97 (7.38)	81.25 (6.62)	0.727
Ethnicity—Non-white/White (%)	90/29 (75.6/24.4)	125/75 (62.5/37.5)	0.022
Male	69 (58.0)	120 (60.0)	0.813
**Comorbidities**			
Ischaemic heart disease (%)	79 (66.4)	117 (58.5)	0.2
Hypertension (%)	80 (67.2)	128 (64.0)	0.643
Dyslipidaemia (%)	45 (37.8)	71 (35.5)	0.768
Diabetes mellitus (%)	31 (26.1)	57 (28.5)	0.731
Previous cancer (%)	27 (22.7)	33 (16.5)	0.223
Active cancer (%)	8 (6.7)	20 (10.0)	0.414
Cognitive impairment (%)	0 (0.0)	11 (5.5)	0.008
COPD (%)	9 (7.6)	18 (9.0)	0.836
Chronic kidney disease (%)	22 (18.5)	42 (21.0)	0.665
Smoking status			0.289
Never smoked (%)	102 (85.7)	111 (79.3)	
Ex-smoker (%)	4 (3.4)	4 (2.9)	
Current smoker (%)	13 (10.9)	25 (17.9)	
**Clinical parameters**			
Haemoglobin	127.00 (16.74)	126.65 (16.76)	0.859
CT calcium score	2727.95 (1655.40)	2509.44 (1332.53)	0.227
**Echocardiographic parameters**			
Aortic valve area (cm)	0.77 (0.30)	0.78 (0.20)	0.674
Mean aortic valve gradient (mmHg)	42.11 (14.02)	39.25 (12.79)	0.098
Peak aortic valve gradient (mmHg)	70.39 (21.68)	66.83 (20.93)	0.209
Aortic velocity (m/s)	4.16 (0.68)	4.07 (0.66)	0.253
Left ventricular ejection fraction (%)	58.46 (8.52)	58.01 (8.60)	0.672
Dimensionless index	0.23 (0.06)	0.23 (0.07)	0.564
Indexed stroke volume (mL)	39.62 (12.37)	39.69 (11.76)	0.967
**Electrocardiographic parameters**			
PR interval duration (ms)	180.84 (47.05)	190.59 (53.07)	0.176
QRS duration (ms)	109.57 (23.23)	107.08 (24.70)	0.41
Bundle branch block = LBBB/RBBB (%)	4/8 (33.3/66.7)	19/17 (52.8/47.2)	0.324
**Outcomes**			
Waiting time to TAVI, days [median (IQR)]	75.00 [38.75, 118.00]	104.00 [61.00, 176.00]	<0.001
Emergency department presentation (%)	16 (13.4)	25 (12.5)	0.863
Unplanned hospitalization (%)	14 (11.8)	21 (10.5)	0.715
Death (%)	3 (2.5)	8 (4.0)	0.752

COPD, chronic obstructive pulmonary disease; IQR, inter-quartile range; LBBB, left bundle branch block; RBBB, right bundle branch block.

^a^ *t*-test; Chi-squared test.

### Propensity score-matched cohort

A matched dataset of 170 patients was created (85 patients in each group), with no significant differences in matched covariates between groups (see Supplementary material online, *Figure S3*) and minimal residual differences in demographic or clinical characteristics between groups (*Table 3*).

**Table 3:** Patient characteristics by group after propensity score-matching

	Control (*n* = 85)	RPM (*n* = 85)	*P*-value^a^
**Demographics**			
Age (SD)	80.32 (7.52)	80.46 (7.43)	0.902
Ethnicity—Non-white (%)	57 (67.1)	62 (72.9)	0.503
Male	46 (54.1)	50 (58.8)	0.643
**Comorbidities**			
Ischaemic heart disease	46 (54.1)	46 (54.1)	1
Hypertension	58 (68.2)	54 (63.5)	0.627
Dyslipidaemia	32 (37.6)	30 (35.3)	0.873
Diabetes mellitus	23 (27.1)	22 (25.9)	1
Previous cancer	17 (20.0)	14 (16.5)	0.691
Active cancer	7 (8.2)	10 (11.8)	0.609
COPD	7 (8.2)	8 (9.4)	1
Chronic kidney disease	15 (17.6)	16 (18.8)	1
Smoking status			0.822
Never smoked	75 (88.2)	75 (88.2)	
Ex-smoker	1 (1.2)	2 (2.4)	
Current smoker	9 (10.6)	8 (9.4)	
**Clinical parameters**			
Haemoglobin	125.80 (17.32)	124.23 (16.82)	0.559
CT calcium score	2679.58 (1370.05)	2383.78 (1371.87)	0.192
**Echocardiographic parameters**			
Aortic valve area (cm)	0.78 (0.33)	0.80 (0.17)	0.728
Mean aortic valve gradient (mmHg)	42.84 (15.68)	37.02 (12.50)	0.017
Peak aortic valve gradient (mmHg)	71.33 (23.74)	65.36 (20.02)	0.125
Aortic velocity (m/s)	4.21 (0.73)	3.98 (0.68)	0.046
Left ventricular ejection fraction (%)	58.62 (8.32)	57.48 (8.92)	0.427
Dimensionless index	0.24 (0.07)	0.24 (0.08)	0.648
Indexed stroke volume (mL)	40.43 (12.15)	40.30 (10.74)	0.95
**Electrocardiographic parameters**			
PR interval duration (ms)	178.02 (45.27)	191.88 (57.49)	0.144
QRS duration (ms)	104.49 (19.66)	105.18 (23.82)	0.848
Bundle branch block = LBBB/RBBB (%)	2/5 (28.6/71.4)	9/3 (75.0/25.0)	0.074
**Outcomes**			
Waiting time to TAVI, days [median (IQR)]	68.00 [27.00–98.00]	55.5 [34.75–101.75]	0.729
Emergency department presentation (%)	12 (14.1)	13 (15.3)	1
Unplanned hospitalization (%)	11 (12.9)	11 (12.9)	1
Death (%)	3 (3.5)	2 (2.4)	1

COPD, chronic obstructive pulmonary disease; IQR, inter-quartile range; LBBB, left bundle branch block; RBBB, right bundle branch block.

^a^ *t*-test; Chi-squared test or Fisher’s exact test.

### Waiting time

As a result of substantial growth of the waiting list, in the unadjusted cohort, the waiting time to TAVI experienced by the RPM group was significantly longer compared with the historical control group (median 104 vs. 75 days, *P* < 0.001). After PSM, there was no significant difference in waiting times between groups (median 55.5 vs. 68.00, *P* = 0.729). The longer waiting times observed in the RPM cohort reflect contextual system pressures during the study period, including sustained growth in TAVI referrals without a corresponding increase in procedural capacity. These operational constraints were unrelated to the RPM protocol itself, which was implemented as an adjunct to existing care processes rather than as a scheduling mechanism.

### Association between remote monitoring and adverse events

During the observation period in the RPM cohort, 25 (13%) patients presented to ED, 21 (11%) patients suffered unplanned hospitalization and there were eight (4%) deaths.

**Figure 3:**
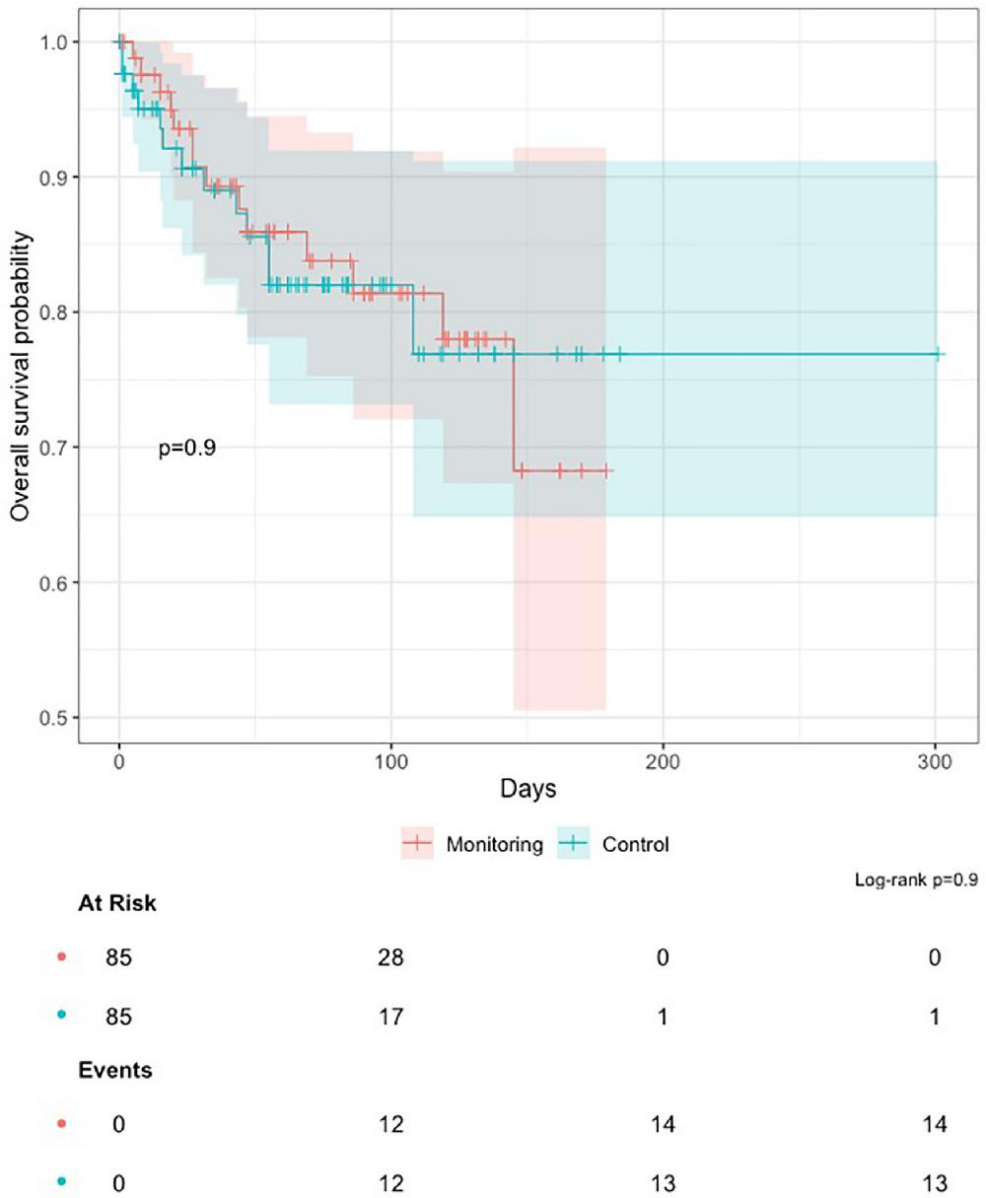
Kaplan-Meier survival curve. Survival probability free from major adverse events in the propensity score-matched RPM (red) and control (blue) groups.

There were no statistically significant differences in rates of ED presentation, unplanned hospitalization or death between RPM and historical control groups at baseline (*Table 2*). This was also observed in the PSM cohort, where no difference in survival probability was identified (log-rank *P* = 0.9, *Figure 3*).

After sensitivity analysis using IPTW applied to all eligible patients (see Supplementary material online, *Table S4*), results were concordant with the PSM analysis, with no statistically significant difference in event-free survival between RPM and control groups (IPTW-adjusted Log-rank *P* = 0.939, Supplementary material online, *Figure S4*).

In the sensitivity analysis using multiple imputation with IPTW adjustment, none of the additional covariates (left ventricular ejection fraction, dimensionless index, peak transaortic gradient, aortic valve calcium score) were significantly associated with major adverse cardiovascular events after adjusting for RPM/control allocation (see Supplementary material  *S5*).

### Safety—association between waiting time and major adverse events

**Figure 4:**
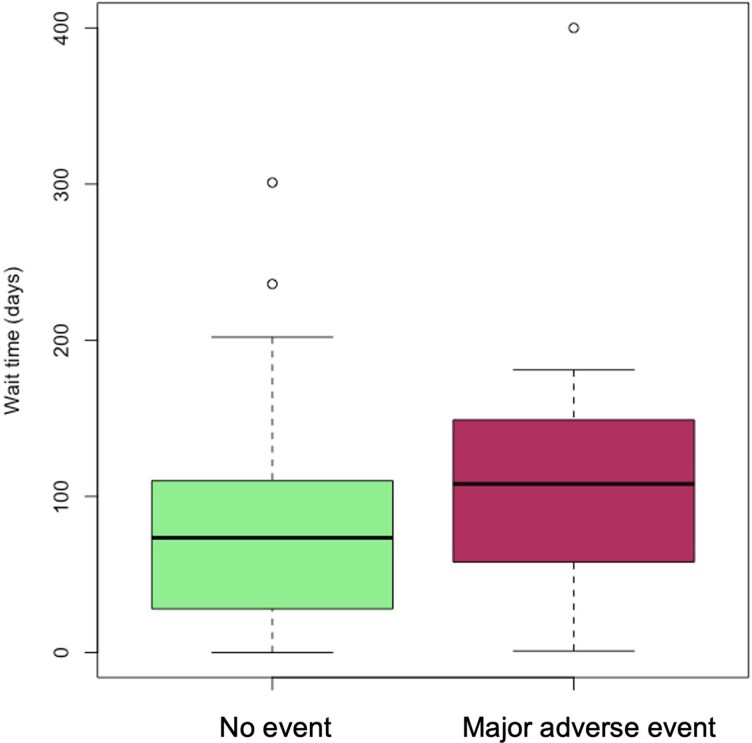
Box plots of waiting times stratified by experience of major adverse events. Box plot partition indicates the median waiting time. Boxes are bounded by the upper (75th) and lower (25th) centile. Error bars denote the minimum (first quartile minus 1.5 × interquartile range) and maximum (third quartile plus 1.5 × interquartile range) limits.

There was a trend towards longer median waiting time to TAVI in patients who suffered an adverse event compared with those who remained event-free throughout their waiting period, but this difference was not significant (111 vs. 78.2 days, *P* = 0.17, *Figure 4*).

In the historical control cohort, there was no significant association between wait time and adverse events on univariate logistic regression (OR 1.01, 95% CI: 0.01–1.83, *P* = 0.07). When examined in 50-day increments, there was a trend towards increasing odds of major adverse events with increasing waiting times, which did not reach statistical significance (*Table 4*). In the full cohort, multivariable logistic regression including RPM/control group and selected covariates did not identify any significant association between waiting time and adverse events (all *P* > 0.05; Supplementary Material  *S5*).

**Table 4:** Univariate logistic regression model for association between 50-day increments in waiting time and major adverse events

Waiting time category (number of patients)	OR (95% CI)	*P*
100 days > wait ≥ 50 days (69)	1.38 (0.31–6.19)	0.7
150 days > wait ≥ 100 days (36)	3.67 (0.78–17.38)	0.1

### Effectiveness—pathway performance characteristics for detection of deterioration

120/200 (60%) patients receiving RPM were escalated from the NWLVH team to the TAVI team for week-on-week symptomatic deterioration. Of these 53 (44.2%) were further escalated.

**Figure 5:**
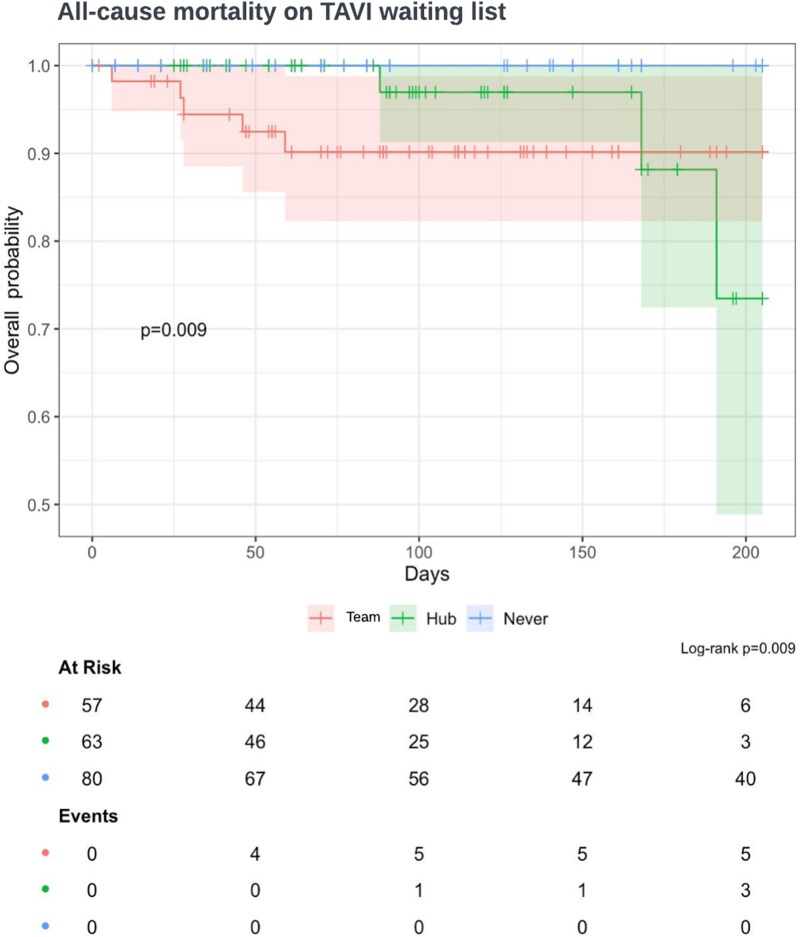
Kaplan-Meier survival curves stratified by alert status of patients receiving RPM. Team, TAVI team; Hub, NWLVH monitoring team; Never, no red or amber alert.

The performance characteristics of each escalation point are described in *Tables 5* and *6*, with survival analysis stratified by escalation status presented in *Figure 5*.

**Table 5:** Pathway performance characteristics at escalation point one

Outcome	Sensitivity	Specificity	PPV	NPV
Death	1.00 (0.67–1.00)	0.40 (0.34–0.47)	0.06 (0.03–0.12)	1.00 (0.95–1.00)
Unplanned hospitalization	0.67 (0.45–0.83)	0.41 (0.34–0.48)	0.12 (0.07–0.19)	0.91 (0.83–0.96)

Performance of the NWLVH monitoring team to detect patient deterioration when receiving inbound symptom data from patients receiving RPM.

**Table 6:** Pathway performance characteristics at escalation point two

Outcome	Sensitivity	Specificity	PPV	NPV
Death	0.57 (0.25–0.84)	0.53 (0.44–0.62)	0.07 (0.03–0.17)	0.95 (0.87–0.98)
Unplanned hospitalization	0.57 (0.32–0.79)	0.54 (0.44–0.63)	0.14 (0.07–0.25)	0.91 (0.81–0.96)

Performance of the TAVI team to detect patient deterioration when receiving escalations of potentially deteriorating patients from the NWLVH monitoring team.

Escalation from the NWLVH team to the TAVI team based on detection of week-on-week symptom deterioration (escalation point one) demonstrated high sensitivity for waiting list death and unplanned hospitalization, with low positive predictive value (0.06 and 0.12, respectively). Escalation point two (TAVI team decision to schedule senior TAVI physician review, expedite TAVI date or admit the patient to hospital) demonstrated a moderate sensitivity for waiting list death and unplanned hospitalization (0.57), and high negative predictive value (>0.90).

Subgroup analysis of patients who were escalated to the TAVI team demonstrated that waiting times to TAVI were influenced by escalation decisions (*Table 7*).

**Table 7:** Outcomes of patients escalated to the TAVI team, based on highest grade of response

TAVI CNS Escalation Decisions	Number (%)	Unplanned hospitalization (%)	Death (%)	Median wait time to TAVI from escalation decision to procedure (days)	Median total wait time to TAVI from waiting list placement to procedure (days)
**Total**	**120**	**9 (7.5)**	**8 (6.7)**	**96**	**96**
Reassure	67 (55.8)	6 (9.0)	3 (4.5)	96	96
Expedite TAVI date	22 (18.3)	1 (4.5)	1 (4.5)	25	90
Admit to hospital	5 (4.2)	—	2 (40)	9	28
Senior TAVI Physician review	26 (21.6)	2 (7.7)	2 (7.7)	35	117
*Reassure*	*5* (*19.2)*	*0*	*0*	*85*	*189*
*Expedite TAVI date*	*18* (*69.2)*	*1* (*3.8)*	*1* (*3.8)*	*35*	*121*
*Admit to hospital*	*2* (*7.7)*	*—*	*0*	*12.5*	*59*
*Awaiting review at time of event*	*1* (*3.8)*	*1* (*3.8)*	*1* (*3.8)*	—	—

Values in bold indicate totals

### Digital inclusion

122 out of 200 (61%) patients were able to register with the online platform and complete at least one symptom questionnaire. A further 11 (6%) were supported to do so with the assistance of family or friends visiting them at home. 67 (34%) of patients could not register with the online platform, and received telephone-based symptom monitoring.

There were no significant associations between digital inclusion and age, sex or ethnicity of participants (*Table 8*).

**Table 8:** Univariate logistic regression model for association between patient characteristics and digital inclusion

Characteristic	OR (95% CI)	*P*
Age	0.99 (0.94–1.03)	0.7
Male sex	1.04 (0.59–1.88)	0.9
White ethnicity	0.66 (0.36–1.18)	0.2

95% CI, 95% confidence interval; OR, odds ratio.

## Discussion

Our findings indicate that in the context of substantially longer waiting times to TAVI compared with historical controls (over 30 days’ difference between groups), the implementation of an RPM-guided prioritization pathway was feasible and identified deteriorating patients with high sensitivity. We did not observe increased waiting list mortality during the observation period, which would otherwise have been expected.^13^

### Implications for clinicians and policymakers

Over 40% of centres participating in the European TAVI Pathway Registry report a mean waiting time of greater than or equal to two months.^14^ In the United Kingdom, an estimated 300 000 people have severe aortic stenosis, of which 51 000 are estimated to be eligible for TAVI.^15^ This demand exceeds the current annual operating capacity of the national TAVI programme by more than sevenfold.^2^ As waiting time increases, the cost-effectiveness of TAVI declines, such that substantial reduction of waiting lists is economically beneficial to health systems.^3^ This poses a multifaceted challenge to any national healthcare system in improving and maximizing the efficient use of capacity for TAVI at regional performing centres.^5^

Our findings suggest that RPM can serve not only as a triage tool but also a means to support patients during the waiting period. By embedding the pathway within existing local remote care infrastructure, the pathway minimized additional burden on the TAVI CNS team by assigning first-line triage responsibilities to a generalist monitoring service.

To our knowledge, our study includes one of the oldest patient populations to receive remote monitoring in any published cardiovascular cohort, with a median age of 81 years. Importantly, over 65% of patients accessed the RPM platform through a graded support process. This compares favourably with national data indicating that 29% of UK adults aged over 75 years old do not use the internet at all, and 49% are unable to complete basic digital tasks such as turning on a device or connecting to Wi-Fi.^16^ Our findings underscore the importance of tailored support structures to promote equitable access and facilitate broader implementation of our operational model to other high-risk elective waiting lists.

### Association between waiting time and adverse events

An increase in cumulative adverse events with longer waiting times is expected in this high-risk population, even in the best-case scenario, given the substantial background morbidity of the population. Detecting this association is further complicated by the fact that patients undergoing unplanned procedures have shorter waits, limiting follow-up duration for those at highest risk. Overall, we failed to demonstrate a significant association between waiting time and adverse events (OR 1.01, 95% CI: 0.01–1.83, *P* = 0.07). While our study was not powered to definitively establish an association between waiting time and adverse events, our primary objective was to assess the feasibility, safety, and real-world performance of an RPM-guided prioritization pathway, thereby addressing the implementation hypothesis of mitigating waiting list risk rather than re-examining the aetiological link between waiting time and harm already demonstrated in larger registry studies (>4000 patients).^1^ In our study, there was substantial overlap between patients with ED presentations and unplanned hospitalization, and patients who experienced an adverse event had longer median waiting times to TAVI (111 vs. 78.2 days)—increasing the likelihood of an underlying association that did not reach significance due to the low number of events.

### Impact of prioritization based on RPM

We report a 100% sensitivity for prediction of waiting list death at escalation one (from NWLVH to TAVI team), and a moderate (67%) sensitivity for prediction of unplanned hospitalization. There was a low PPV owing to the substantially high false-positive rate and the low number of events. This highlights a trade-off between the specialist workforce burden associated with high rates of escalation, and the risk of reducing the sensitivity that can be achieved by a non-specialist, ‘first filter’ (NWLVH team) adopting a higher escalation threshold.

As expected, expedited procedures or hospital admissions were associated with shorter times from escalation to procedure, reflecting the capacity of the pathway to bring forward treatment once deterioration was recognized, with the shortest waiting times observed in patients directly admitted to hospital by the TAVI team (median 9 days). In some cases, these shorter post-escalation intervals coincided with shorter total waits, consistent with prioritization decisions made relatively early in the waiting period. This aligns with existing evidence that earlier identification and intervention in high-risk patients may confer the greatest clinical benefit.^3^ However, our data also show instances where escalation occurred later in the waiting period despite rapid scheduling thereafter, suggesting that the timing of deterioration and escalation was heterogeneous across the cohort. This likely reflects a combination of individual disease trajectories and operational constraints, rather than a uniform pattern of disease progression. There was a 40% mortality rate (two out of five patients) amongst patients who received immediate facilitated hospital admission following an escalation to the TAVI team, suggesting that the highest risk patients were correctly identified, but did not receive timely intervention to prevent harm. This is further suggested by the 35-day median wait time to TAVI following the decision to expedite the procedural date—in itself longer than the four weeks nationally-recommended maximum waiting period.^17^ This highlights the scale of the challenge in implementing risk management pathways for cardiac procedural waiting lists in low-capacity settings.

Only one study has examined a risk prediction model for patients awaiting TAVI in a similarly low-capacity setting. Miranda *et al*.^18^ recently reported the derivation and validation of the Canadian TAVI Triage Tool (CAN3T).^18^ They performed a retrospective observational cohort study of 13 380 patients referred for TAVI in Ontario, Canada. The median wait time to TAVI was 78 days [IQR 32–143], which was comparable with the historical control cohort in this study [median 74 days (IQR 38.75–118.00)]. They reported comparable rates of waiting list death (4.5% vs. 2.5% in our study), with substantially higher rates of unplanned hospitalization (33.1% vs. 11.8% in our study). Miranda *et al.*^18^ identified patient baseline clinical variables (e.g. presence of chronic obstructive pulmonary disease, renal disease) that contributed to moderate model performance for prediction of waiting list death [AUC 0.66 (95% CI: 0.63–0.69)]. The model requires external validation to determine its generalizability. There are also substantial challenges to scalable implementation of such a model, such as the need for integration with the electronic health record to enable automated input of model variables. Furthermore, it does not account for longitudinal changes in patient symptoms or vital signs—which can feasibly be captured through RPM. The addition of these variables might improve model performance.

### Limitations

Our findings are best understood in the context of the limitations of this study. First, the single-centre observational study design. In the context of a low absolute event rate, this design restricts the conclusions that can be drawn about the impact of RPM on adverse events. Second, the absence of randomization leaves the interpretation of the results vulnerable to confounding. We attempted to mitigate this by performing propensity score-matching on a wide range of clinical and demographic covariates. However, use of PSM to overcome confounding is limited by the availability and completeness of covariate data. This study used real-world data recorded from the electronic health record of a tertiary centre receiving referrals for TAVI from several referring centres. Therefore, the availability of certain data e.g. echocardiographic parameters was mixed. Therefore, we could not include these covariates in the logistic regression model without prohibitively restricting the size of the resultant matched cohort. However, there were minimal differences between the RPM and historical control cohorts at baseline, enhancing the generalizability of our findings. Furthermore, we examined a regional health record (LCR) for primary outcome assessment, mitigating the recall bias associated with patient surveys or unstructured outcome data recorded in individual medical note entries. This is particularly important in the context of a regional TAVI programme, since patients on the waiting list are more likely to experience emergency presentations or unplanned admissions to their local hospital.

A formal implementation study design, involving longitudinal examination of system and patient acceptability and adaptation data, would enable a deeper understanding of the feasibility and transportability of the intervention. While the high escalation sensitivity suggests clinical viability, we did not capture structured process or implementation outcomes such as fidelity, adaptation or workforce burden. This limits the interpretability of feasibility findings and should be addressed in future studies designed with an explicit implementation science framework.^19^ Finally, the implementation could not control for contextual factors related to the health system. For example, the addition of weekend elective TAVI procedural lists; cardiac catheterization lab utilization rates; procedural cancellation rates would all impact variably on capacity during the observation period and hence influence waiting times. The symptom questionnaire focused on classical symptoms of severe aortic stenosis. However, in older adults, deterioration may present atypically or manifest through behavioural adjustment, such as reduced physical exertion.^20^ These patterns of change may not have been fully captured through the symptom questionnaire, and highlights the potential for future iterative co-development and validation of the questionnaire with patient partners. It is also possible that symptom deterioration was conveyed through alternative communication channels, such as telephone calls to the Cardiology service or in routine outpatient clinic reviews—which were not captured by the study protocol but may have informed prioritization decisions. While these limitations may have attenuated the completeness of symptom surveillance, they reflect the complexity of real-world care pathways. However, we investigated the associations between clinical decision-making and outcomes in a contemporary, real-world setting. Consequently, our findings can directly inform training needs, escalation thresholds and capacity planning for wider implementation of RPM for patients waiting for TAVI.

## Conclusion

RPM for patients awaiting TAVI can potentially mitigate the adverse effects of longer waiting times through accurate detection of deterioration, and by informing prioritization decisions.

## Supplementary Material

ztaf114_Supplementary_Data

## Data Availability

Data are available upon reasonable request to the corresponding author.
