# Supplementary material for: Safety and effectiveness of remote monitoring and prioritization of patients awaiting transcatheter aortic valve implantation: a propensity-matched prospective observational cohort study
Source: Eur Heart J Digit Health. 2025 Oct 3;7(2):ztaf114. doi: 10.1093/ehjdh/ztaf114 (PMC12862160; doi:10.1093/ehjdh/ztaf114)
Supplement: ztaf114_Supplementary_Data [file ztaf114_supplementary_data.docx]

**Supplementary Material**

**Supplementary Table 1**

STROBE Statement—checklist of items that should be included in reports of observational studies

|  | **Item No.** | **Recommendation** | **Page No.** | **Relevant text from manuscript** |
| --- | --- | --- | --- | --- |
| **Title and abstract** | 1 | (*a*) Indicate the study’s design with a commonly used term in the title or the abstract | 1,2 | ‘Prospective observational |
|  |  |  |  | cohort study’ as stated in |
|  |  |  |  | the title on page 1 and the |
|  |  |  |  | abstract on page 2 |
|  |  | (*b*) Provide in the abstract an informative and balanced summary of what was done and what was found | 2 | Provided in Abstract on |
|  |  |  |  | page 2 |
| **Introduction** |  |  |  |  |
| Background/rationale | 2 | Explain the scientific background and rationale for the investigation being reported | 3 | Included in the |
|  |  |  |  | introduction on page 3: |
|  |  |  |  | ‘Procedural waiting times |
|  |  |  |  | at UK regional TAVI |
|  |  |  |  | centres are not routinely |
|  |  |  |  | reported, however they |
|  |  |  |  | are invariably longer than |
|  |  |  |  | the recommended |
|  |  |  |  | national upper limit of four |
|  |  |  |  | weeks5, with a growing |
|  |  |  |  | proportion of TAVIs |
|  |  |  |  | performed as urgent or |
|  |  |  |  | emergency procedures2. |
|  |  |  |  | Mitigating risk from longer |
|  |  |  |  | waiting times is therefore |
|  |  |  |  | a priority in low-capacity |
|  |  |  |  | settings relative to the |
|  |  |  |  | needs of the population.’ |
| Objectives | 3 | State specific objectives, including any prespecified hypotheses | 3 | Included in the |
|  |  |  |  | introduction on page 3: ‘In |

this study, we aimed to test the hypotheses that implementing a DHT- enabled remote monitoring and prioritisation pathway for patients on the TAVI waiting list is feasible, safely reduces the risk of waiting list death in the context of escalating waiting times, can effectively identify patients at risk of waiting list deterioration, and therefore inform prioritisation decisions.’

**Methods**

Study design 4 Present key elements of study design early in the paper 4, 5 & 6

Included in the Methods page 4, 5 & 6.

Setting 5 Describe the setting, locations, and relevant dates, including periods of recruitment, exposure, follow-up, and data collection 6, 7 Included in the Methods

page 6 & 7: ‘All patients prospectively added to the waiting list between 24th April 2023 and 15th November 2023.’

Participants 6 (*a*) *Cohort study*—Give the eligibility criteria, and the sources and methods of selection of participants. Describe methods of follow-up

*Case-control study*—Give the eligibility criteria, and the sources and methods of case ascertainment and control selection. Give the rationale for the choice of cases and controls

*Cross-sectional study*—Give the eligibility criteria, and the sources and methods of selection of participants

(*b*) *Cohort study*—For matched studies, give matching criteria and number of exposed and unexposed

*Case-control study*—For matched studies, give matching criteria and the number of controls per case

4,5 Cohort study – Included in

Methods page 4 & 5

Variables 7 Clearly define all outcomes, exposures, predictors, potential confounders, and effect modifiers. Give diagnostic criteria, if applicable 7, 8

& 9

Included in the Methods page 7, 8 & 9 under subheadings Outcomes,

|  | | | | Bias and Statistical  analysis |
| --- | --- | --- | --- | --- |
| Data sources/  measurement | 8* | For each variable of interest, give sources of data and details of methods of assessment (measurement). Describe comparability of  assessment methods if there is more than one group | 4-7 | Included in the Methods  page 4-7 |
| Bias | 9 | Describe any efforts to address potential sources of bias | 7,8 | Included in the Methods  page 7,8 under subheading Bias |
| Study size | 10 | Explain how the study size was arrived at | 4,5 | Included in the Methods page 4,5 under  subheading Bias |
| Continued on next page |  |  |  |  |

| Quantitative  variables | 11 | Explain how quantitative variables were handled in the analyses. If applicable, describe which groupings were  chosen and why | 8,9 | Included in Methods page 8,9 under  subheading statistical analysis |
| --- | --- | --- | --- | --- |
| Statistical  methods | 12 | (*a*) Describe all statistical methods, including those used to control for confounding | 8,9 | Included in Methods page 8,9 under  subheading statistical analysis |
|  |  | (*b*) Describe any methods used to examine subgroups and interactions | 8,9 | Included in Methods page 8,9 under  subheading statistical analysis |
|  |  | (*c*) Explain how missing data were addressed | 8,9 | Included in Methods page 8,9 under  subheading statistical analysis |
|  |  | (*d*) *Cohort study*—If applicable, explain how loss to follow-up was addressed  *Case-control study*—If applicable, explain how matching of cases and controls was addressed  *Cross-sectional study*—If applicable, describe analytical methods taking account of sampling strategy | 4 | Included in Methods page 4 |
|  |  | (*e*) Describe any sensitivity analyses | - | Not performed |
| **Results** |  |  |  |  |
| Participants | 13* | (a) Report numbers of individuals at each stage of study—eg numbers potentially eligible, examined for eligibility,  confirmed eligible, included in the study, completing follow-up, and analysed | 10 | Included in Results page 10, Figure 2. |
|  |  | (b) Give reasons for non-participation at each stage | 10 | Figure 2 |
|  |  | (c) Consider use of a flow diagram | 10 | Figure 2 |
| Descriptive data | 14* | (a) Give characteristics of study participants (eg demographic, clinical, social) and information on exposures and  potential confounders | 10-13 | Included in Results page 10-13, Tables  2 & 3 |
|  |  | (b) Indicate number of participants with missing data for each variable of interest | 10-13 | Included in Results page 10-13, Tables  2 & 3 |
|  |  | (c) *Cohort study*—Summarise follow-up time (eg, average and total amount) | 10 | Included in Results page 10: |
| Outcome data | 15* | *Cohort study*—Report numbers of outcome events or summary measures over time | 10-13 | Included in Results page 10-13, Tables  2 & 3 |
|  |  | *Case-control study—*Report numbers in each exposure category, or summary measures of exposure |  |  |
|  |  | *Cross-sectional study—*Report numbers of outcome events or summary measures |  |  |
| Main results | 16 | (*a*) Give unadjusted estimates and, if applicable, confounder-adjusted estimates and their precision (eg, 95%  confidence interval). Make clear which confounders were adjusted for and why they were included | 10-15 | Included in Results page 10-15 |
|  |  | (*b*) Report category boundaries when continuous variables were categorized | 10-13 | Included in Results page 10-13 |
|  |  | (*c*) If relevant, consider translating estimates of relative risk into absolute risk for a meaningful time period |  |  |
| Continued on next page |  |  |  |  |

Other analyses 17 Report other analyses done—eg analyses of subgroups and interactions, and sensitivity analyses

**Discussion**

Key results 18 Summarise key results with reference to study objectives 22 Included in discussion on page 22: ‘Our findings indicate that in the context of substantially longer waiting times to TAVI compared to historical controls (over 30 days’ difference between groups), the implementation of an RPM-guided prioritisation pathway was feasible and identified deteriorating patients with high sensitivity. We did not observe increased waiting list mortality during the observation period, which would otherwise have been expected”

Limitations 19 Discuss limitations of the study, taking into account sources of potential bias or imprecision. Discuss both direction and

magnitude of any potential bias

Interpretation 20 Give a cautious overall interpretation of results considering objectives, limitations, multiplicity of analyses, results from similar studies, and other relevant evidence

24-25

22-23

Generalisability 21 Discuss the generalisability (external validity) of the study results 22-23

**Other information**

Funding 22 Give the source of funding and the role of the funders for the present study and, if applicable, for the original study on 26

which the present article is based

*Give information separately for cases and controls in case-control studies and, if applicable, for exposed and unexposed groups in cohort and cross-sectional studies.

**Supplementary figure 1. Symptom questionnaire and response risk matrix**

| **GREY = no alert**  **AMBER = needs actioning within 2 working days**  **RED = needs actioning within 1 working day**   1. **In the last week, have you experienced any chest pain / ache / tightness / heaviness? (Y/N)**   If Y:  *a) After heavy exertion e.g. playing sport, heavy lifting*  *b) After moderate exertion e.g. climbing stairs quickly, walking in cold weather or in wind*  *c) After mild exertion e.g. climbing one flight of stairs at a normal pace*  *d) At rest*  If Y to (1) proceed to (2)  If N to (1) proceed to (3)   1. **Compared to LAST WEEK, has your chest pain/ache/tightness or heaviness changed? (Y/N)**  - *If Y A) Absent e.g. no chest pain at all during the last week*   *B) The same as week before*  *C) Slightly worse*  *D) Much worse*   1. **How far are you able to walk before chest pain or breathlessness stops you?**  - *Patient selects either: A) Unlimited: 500m +*   *B) 200-500m*  *C) 50-200m*  *D) <50m*  *E) My chest pain is so bad I cannot leave the house*   1. **Over the last week, has your breathlessness been:**  - *Patient selects either: A) Absent e.g. you have not felt breathless at all*   *B) The same as week before*  *C) Slightly worse*  *D) Much worse*   1. **In the last week, have you experienced any ankle swelling?**  - Y/N   ***If yes* Compared to last week, how swollen are your ankles?**   - *Patient selects either: A) My ankles are not swollen*   *B) Same amount of swelling as before*  *C) Slightly worse*  *D) Much worse*  **6. Has there been a change in your water tablet daily dose (Frusemide, Bumetanide….) in the last week? Y/N**   - If Y,: *A) Not taking any water tablets now*   *B) Same dose as usual*  *C) Sometimes taking higher dose than usual*  *D) Consistently more than usual*   1. **Have you felt dizzy or light-headed when you walk during the last week? (Y/N)**   **If Y How bad has your dizziness/light-headedness been compared to the week before?**   - *If Y,: A) Absent e.g. no dizziness at all during the last week*   *B) The same as week before*  *C) Slightly worse*  *D) Much worse*  **8. Have you blacked out/lost consciousness in the last week? (Y/N)**   - (If NO)**: Have you felt close to blacking out/passing out during the last week**? (Y/N) |
| --- |

**Supplementary Table 2**

Missingness of covariates in the pre-matched cohort

| **Variable** | **Missingness** |
| --- | --- |
| **Age** | 0% |
| **Ethnicity** | 0% |
| **Sex** | 0% |
| **IHD** | 0% |
| **Hypertension** | 0% |
| **Dyslipidaemia** | 0% |
| **Diabetes mellitus** | 0% |
| **Previous cancer** | 0% |
| **Active cancer** | 0% |
| **Cognitive impairment** | 0% |
| **COPD** | 0% |
| **Chronic kidney disease** | 0% |
| **Smoking status** | 0% |
| **Hb** | 0% |
| **AVA_cm** | 31% |
| **mean_AVG** | 21% |
| **peak_AVG** | 28% |
| **Aortic_vel** | 10% |
| **LVEF** | 14% |
| **DI** | 36% |
| **strokevol_ind** | 27% |
| **Ca_score** | 10% |
| **PR_int** | 32% |
| **QRS_dur** | 14% |
| **bundlebranch** | 82% |

**Supplementary Table 3**

|  | Type | Control unmatched | RPM unmatched | Mean difference (unmatched) | Control (matched) | RPM (matched) | Mean difference (matched) |
| --- | --- | --- | --- | --- | --- | --- | --- |
| distance | Distance | 0.564 | 0.464 | 0.597 | 0.519 | 0.527 | 0.048 |
| Age | Contin. | 80.975 | 80.098 | 0.124 | 80.318 | 80.459 | 0.02 |
| Sex_Male | Binary | 0.58 | 0.58 | 0.001 | 0.541 | 0.588 | 0.047 |
| Ethnicity_White | Binary | 0.244 | 0.402 | 0.158 | 0.329 | 0.271 | 0.059 |
| IHD | Binary | 0.664 | 0.509 | 0.155 | 0.541 | 0.541 | 0 |
| Hypertension | Binary | 0.672 | 0.634 | 0.038 | 0.682 | 0.635 | 0.047 |
| Dyslipidaemia | Binary | 0.378 | 0.304 | 0.075 | 0.376 | 0.353 | 0.024 |
| Diabetes | Binary | 0.261 | 0.304 | 0.043 | 0.271 | 0.259 | 0.012 |
| Cancer | Binary | 0.261 | 0.205 | 0.055 | 0.235 | 0.247 | 0.012 |
| Cog_Impair | Binary | 0 | 0.045 | 0.045 | 0 | 0 | 0 |
| COPD | Binary | 0.076 | 0.116 | 0.04 | 0.082 | 0.094 | 0.012 |
| CKD | Binary | 0.185 | 0.223 | 0.038 | 0.176 | 0.188 | 0.012 |
| Smoking_0 | Binary | 0.857 | 0.839 | 0.018 | 0.882 | 0.882 | 0 |
| Smoking_1 | Binary | 0.034 | 0.018 | 0.016 | 0.012 | 0.024 | 0.012 |
| Smoking_2 | Binary | 0.109 | 0.143 | 0.034 | 0.106 | 0.094 | 0.012 |

Means of each covariate in the pre-PSM and post-PSM cohorts

**Supplementary Figure 2**


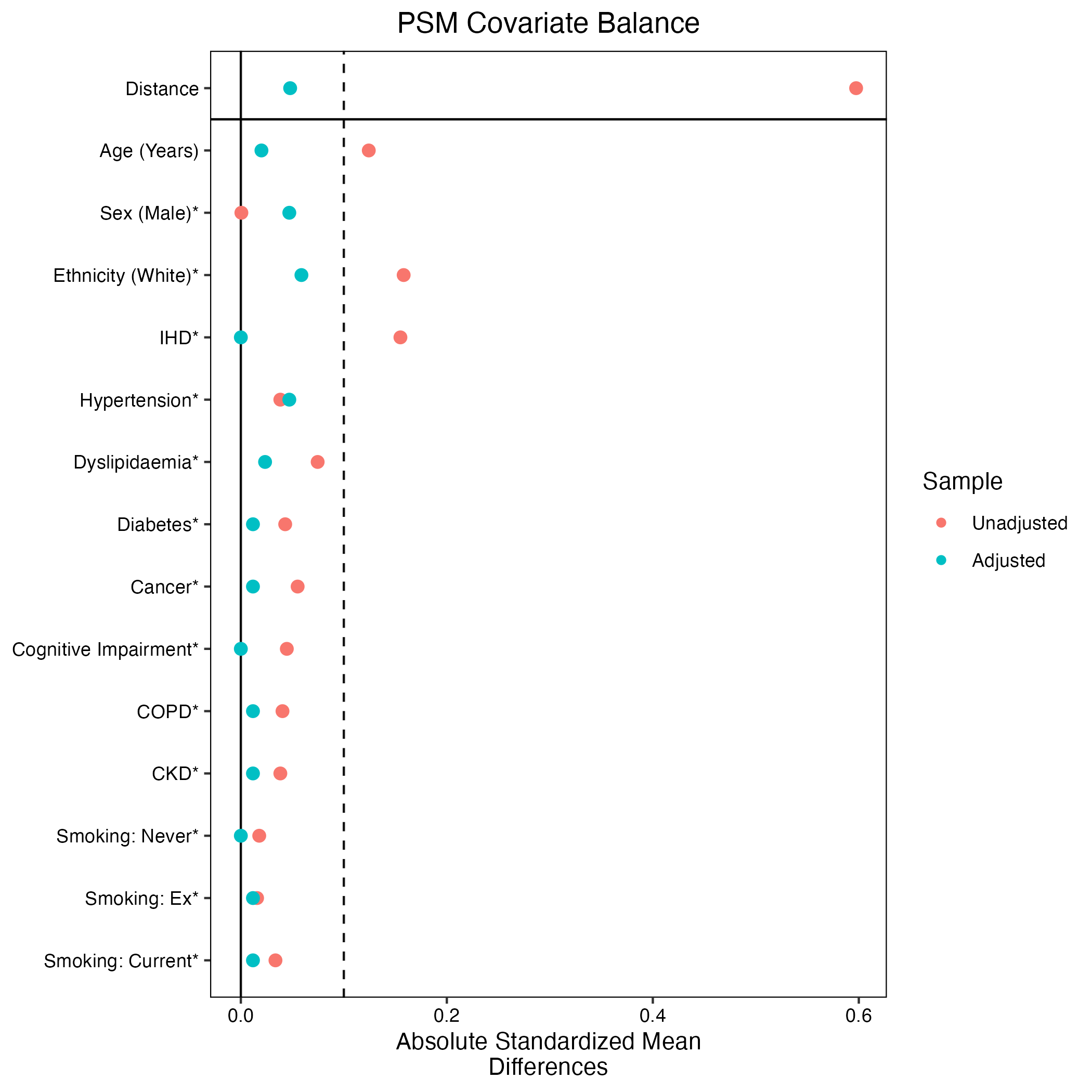


Love plot indicating standardised mean differences between adjusted and unadjusted groups following propensity score-matching

**Supplementary Table 4**

|  | Type | RPM (Unadjusted) | Control (Unadjusted) | Difference (unadjusted) | RPM adjusted | Control adjusted | Difference (adjusted) | Matching Threshold |
| --- | --- | --- | --- | --- | --- | --- | --- | --- |
| prop.score | Distance | 0.544 | 0.429 | 0.78 | 0.544 | 0.523 | 0.139 |  |
| Age | Contin. | 80.098 | 80.975 | 0.119 | 80.098 | 79.596 | 0.068 | Balanced, <0.1 |
| Sex_Male | Binary | 0.58 | 0.58 | 0.001 | 0.58 | 0.566 | 0.014 | Balanced, <0.1 |
| Ethnicity_White | Binary | 0.402 | 0.244 | 0.158 | 0.402 | 0.387 | 0.015 | Balanced, <0.1 |
| IHD | Binary | 0.509 | 0.664 | 0.155 | 0.509 | 0.5 | 0.009 | Balanced, <0.1 |
| Hypertension | Binary | 0.634 | 0.672 | 0.038 | 0.634 | 0.603 | 0.031 | Balanced, <0.1 |
| Dyslipidaemia | Binary | 0.304 | 0.378 | 0.075 | 0.304 | 0.341 | 0.038 | Balanced, <0.1 |
| Diabetes | Binary | 0.304 | 0.261 | 0.043 | 0.304 | 0.254 | 0.05 | Balanced, <0.1 |
| Cancer | Binary | 0.205 | 0.261 | 0.055 | 0.205 | 0.224 | 0.019 | Balanced, <0.1 |
| Cog_Impair | Binary | 0.045 | 0 | 0.045 | 0.045 | 0 | 0.045 | Balanced, <0.1 |
| COPD | Binary | 0.116 | 0.076 | 0.04 | 0.116 | 0.127 | 0.011 | Balanced, <0.1 |
| CKD | Binary | 0.223 | 0.185 | 0.038 | 0.223 | 0.216 | 0.007 | Balanced, <0.1 |
| Smoking_0 | Binary | 0.839 | 0.857 | 0.018 | 0.839 | 0.853 | 0.014 | Balanced, <0.1 |
| Smoking_1 | Binary | 0.018 | 0.034 | 0.016 | 0.018 | 0.019 | 0.001 | Balanced, <0.1 |
| Smoking_2 | Binary | 0.143 | 0.109 | 0.034 | 0.143 | 0.128 | 0.015 | Balanced, <0.1 |

Means of each covariate in the pre-IPTW and post-IPTW cohorts

**Supplementary Figure 3**


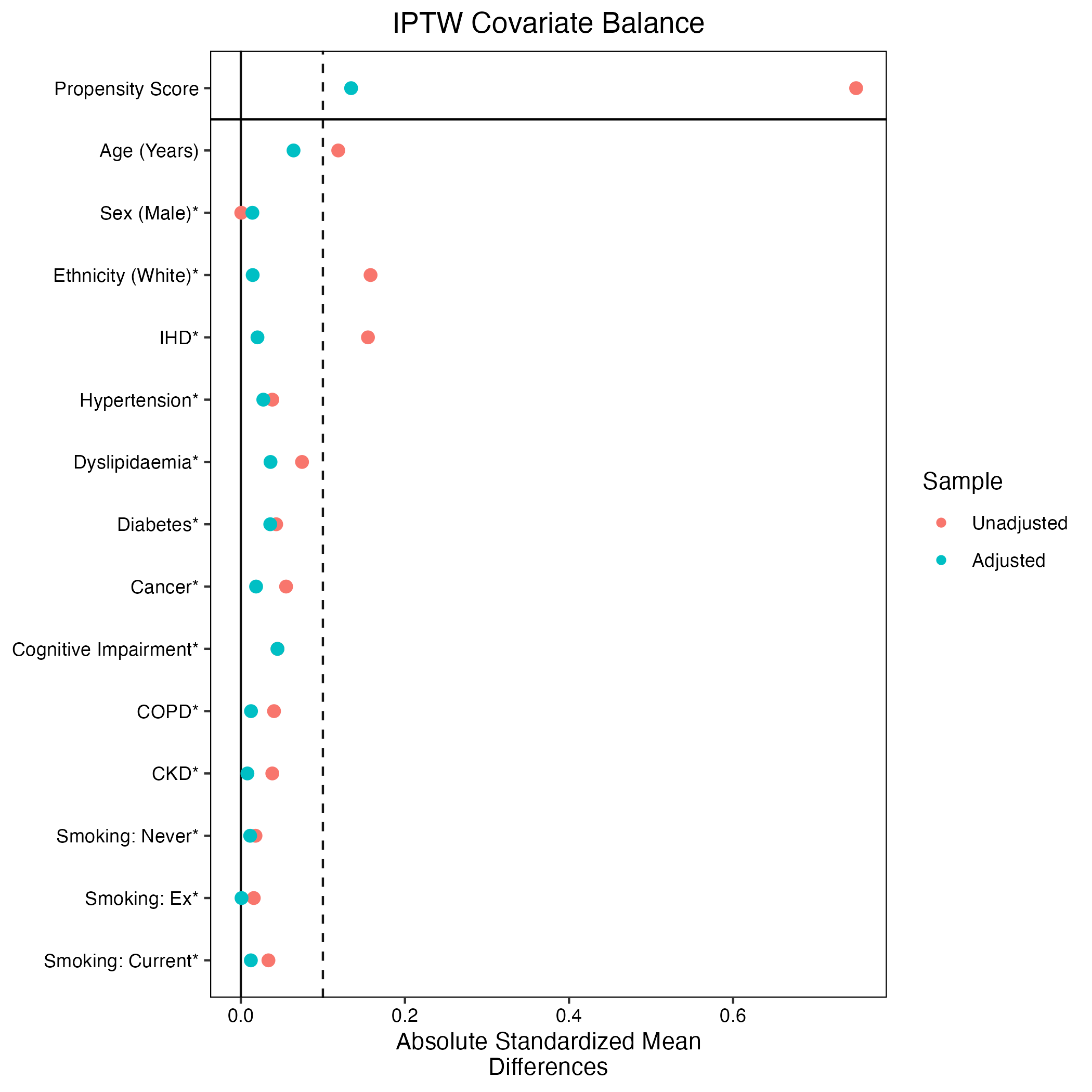


Love plot indicating standardised mean differences between adjusted and unadjusted groups following inverse probability of treatment weighting

**Supplementary Figure 4**


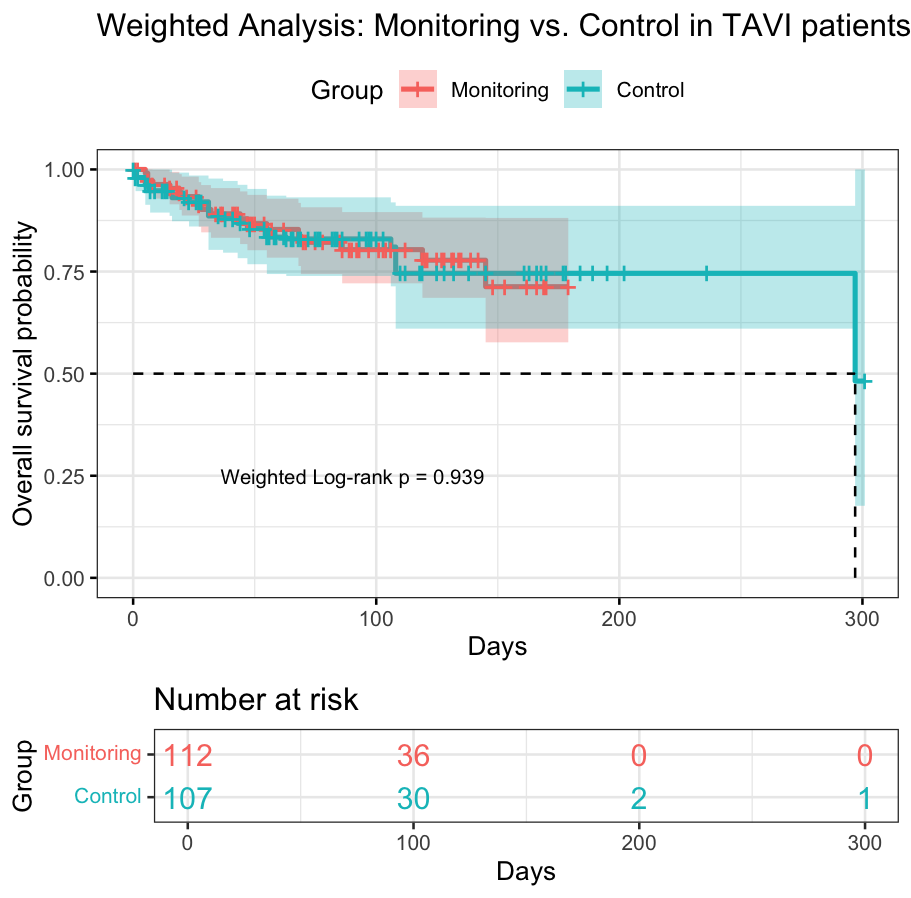


(Sensitivity) Kaplan-Meier analysis using the IPTW cohort with weighted log-rank.

**Supplementary Material 5.**

Logistic regression models for association between covariates and major adverse cardiovascular events following multiple imputation by chained equations (MICE) and IPTW adjustment.

| **Term** | **Estimate** | **Std. Error** | **Statistic** | **df** | **p-value** | **2.5 % CI** | **97.5 % CI** |
| --- | --- | --- | --- | --- | --- | --- | --- |
| **Model 1: LVEF + DI** |  |  |  |  |  |  |  |
| Intercept | -0.516 | 1.226 | -0.420 | 173.445 | 0.674 | -2.935 | 1.903 |
| Control group | -0.007 | 0.407 | -0.019 | 209.390 | 0.985 | -0.810 | 0.794 |
| LVEF | 0.0347 | 0.0214 | 1.628 | 178.011 | 0.105 | -0.007 | 0.076 |
| DI | 0.667 | 3.756 | 0.178 | 108.533 | 0.859 | -6.777 | 8.110 |
| **Model 2: Aortic valve calcium score + Peak Gradient** |  |  |  |  |  |  |  |
| Intercept | -0.387 | 1.173 | -0.330 | 201.686 | 0.742 | -2.701 | 1.926 |
| Control group | -0.0049 | 0.407 | -0.012 | 204.742 | 0.990 | -0.806 | 0.796 |
| Ao Ca score | 0.0358 | 0.0206 | 1.740 | 182.764 | 0.084 | -0.0048 | 0.076 |
| Peak Aortic Gradient | -0.00043 | 0.0087 | -0.049 | 107.002 | 0.961 | -0.0177 | 0.0168 |


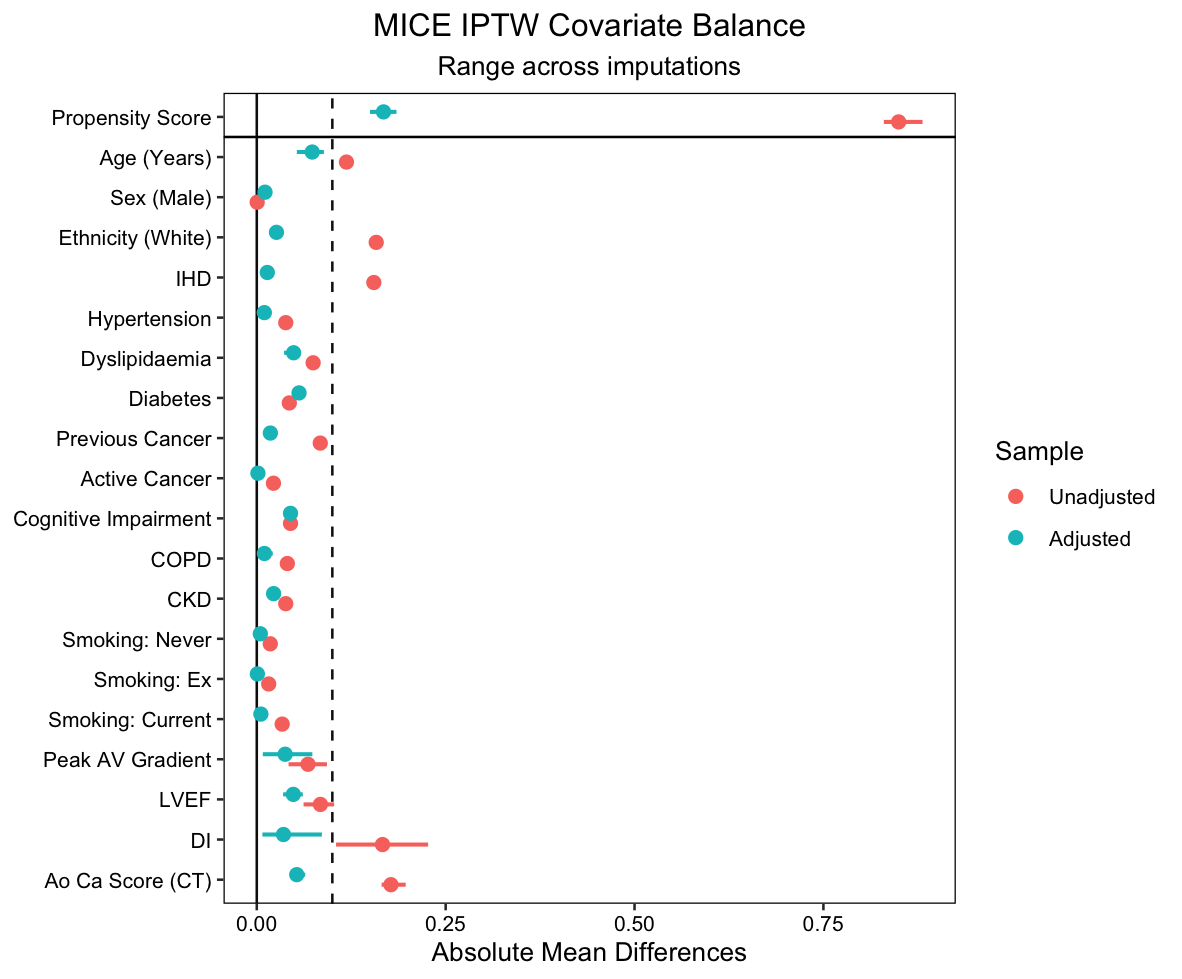


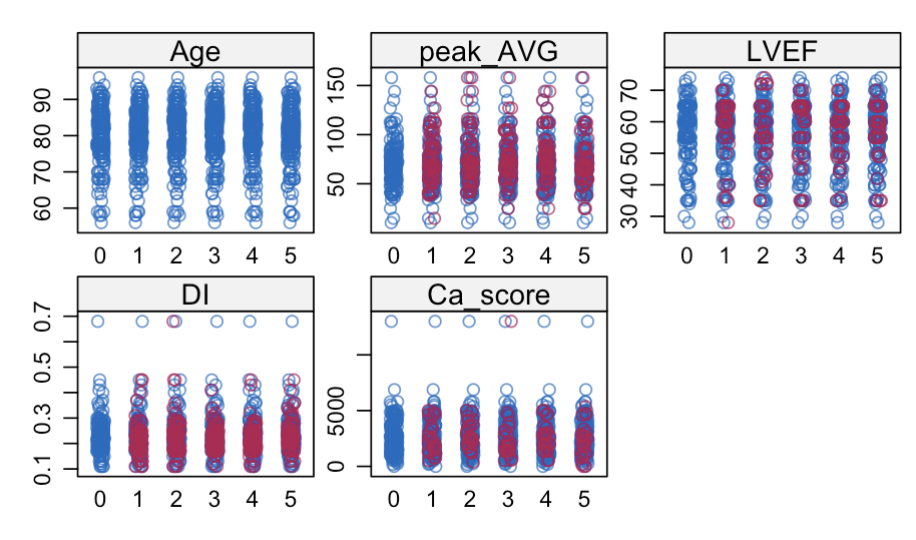


**MICE - 20 cycles with 5 imputed datasets per cycle. Strip plot of observed values (blue) and imputed values (red)**

**Supplementary Material 6**

Multivariable logistic regression for association between waiting time and major adverse cardiovascular events (MACE) in the full cohort (n=36 events).

| **Model** | **Term** | **Estimate** | **Std. Error** | **z value** | **p-value** |
| --- | --- | --- | --- | --- | --- |
| **1: Wait time, age, sex** | Intercept | 2.0737 | 2.0545 | 1.009 | 0.313 |
|  | Wait time (days) | -0.00227 | 0.00311 | -0.730 | 0.465 |
|  | Age (years) | -0.00378 | 0.02524 | -0.150 | 0.881 |
|  | Male sex | 0.18809 | 0.37887 | 0.496 | 0.620 |
| **2: Wait time, group, age** | Intercept | 2.0790 | 2.0659 | 1.006 | 0.314 |
|  | Wait time (days) | -0.00203 | 0.00305 | -0.665 | 0.506 |
|  | Group (control=0) | 0.22305 | 0.36562 | 0.610 | 0.542 |
|  | Age (years) | -0.00414 | 0.02541 | -0.163 | 0.870 |
